# A preliminary evaluation of the training effects of a didactic and simulation-based psychological first aid program in students and school counselors in South Korea

**DOI:** 10.1371/journal.pone.0181271

**Published:** 2017-07-17

**Authors:** Jong-Sun Lee, Sungeun You, Yun-Kyeung Choi, Hyae-young Youn, Hye Sook Shin

**Affiliations:** 1 Department of Psychology, Kangwon National University, Chuncheon, Kangwon, Korea; 2 Department of Psychology, Chungbuk National University, Cheongju, Chungbuk, Korea; 3 Department of Psychology, Keimyung University, Daegue, Korea; 4 Department of Education, Kangwon National University, Chuncheon, Kangwon, Korea; Waseda University, JAPAN

## Abstract

The present study aimed to examine the training effects of a didactic and simulation-based psychological first aid (PFA) program. Based on the competency-based model, the study sought to examine whether the PFA training would enhance knowledge, skills, and attitudes. Study 1 examined the training effects of the PFA program in a sample of undergraduate and graduate students in psychology. Study 2 was conducted with school counselors. In both studies, all participants completed a one-day PFA workshop with a 3-hour didactic lecture and a 3-hour simulation-based practice. Assessments were conducted prior to the didactic lecture and upon completion of the simulation-based practice. In study 1, an examination of pre- and posttest comparisons indicated that the training significantly improved students’ PFA knowledge and perceived competence in PFA skill. In study 2, the same PFA training significantly improved school counselors’ PFA knowledge, perceived competence in PFA skill, perceived preparedness and confidence to provide psychological assistance for future disasters, but their perceived willingness to participate in psychological assistance did not significantly change after the training. This study provides preliminary evidence supporting the effectiveness of the PFA training program using a combined method of didactic and simulation-based practice for disaster mental health providers in Korea.

## Introduction

Psychological first aid (PFA) is an evidence-informed, consensus-driven approach of early intervention following disasters [1]. PFA aims at helping survivors promote a sense of safety and comfort, reduce acute stress responses, increase adaptive coping, and connect to community resources and support systems [2–4]. The World Health Organization (WHO), National Child Traumatic Stress Network (NCTSN), American Red Cross, and other related organizations provide PFA training programs. Not only mental health professionals, but first responders or lay community people are also eligible to provide PFA if they complete relevant training. Indeed, it is necessary that all individuals who work with survivors get PFA training prior to their initial contact with survivors [5].

Although mental health professionals have recommended PFA as an early intervention for disaster survivors, empirical evidence for the effectiveness of PFA interventions is insufficient [6–8]. This may be due to difficulty conducting research in the acute phase of disasters. Researchers have attempted to provide evidence-based guidelines and principles using expert panels, the Delphi process, or available evidence from literature searches [9–11]. Based on these guidelines and principles, various forms of PFA training are available worldwide. Allen and colleagues [12] investigated the perceptions of providers responding to Hurricanes Gustav and Ike among those who received PFA training provided by the NCTSN, and reported an increased level of participants’ perceived confidence in working with survivors. Yet, Fox and collegues [8] reported that scientific evidence for psychological first aid is in the category of “evidence-informed” and lacking proof of training effectiveness based on their examination of peer-reviewed literature from 1990 to 2010.

McCabe and colleagues [1] suggested a competency-based training model of PFA emphasizing three domains of the learning process: cognitive (knowledge), psychomotor (skills), and affective (attitudes). Consistent with this competency-based model, the Johns Hopkins RAPID-PFA program was developed [13]. In the RAPID-PFA program, a combined method of lecture and small group activities was implemented to address five core components of PFA: reflective listening, assessment, prioritization, intervention, disposition, and self-care. Everly et al.[13] reported that RAPID-PFA training improved knowledge, self-efficacy in application of interventions, and confidence in personal resilience for non-mental health trained public health personnel.

Simulation-based training has been reported to be beneficial for knowledge transfer and skill acquisition in medical education [14]. In particular, role-play provides an opportunity to ‘put oneself in someone else’s shoes,’ and help trainees increase their understanding of the patient’s view [14]. Such simulation-based training has been expanded to disaster mental health training [15–17]. In a study with nursing students, Alim, Kawabata, and Nakazawa [15] reported significant improvement in knowledge and observed skills using a pre- and posttest design following in-class training and a disaster drill.

Responding to and meeting disaster-related psychological needs are being increasingly recognized in Korea, especially due to recent nationwide disasters such as the Sewol ferry disaster that occurred in 2014 and a series of earthquakes since 2016; thus, training qualified PFA providers is of great importance. In Korea, the Korean Red Cross runs disaster psychological recovery and support centers in 17 administrative districts in support of the Korean Ministry of Public Safety and Security. Also, community mental health centers run by the Korean Ministry of Health and Welfare provide psychosocial support in times of disasters. Yet, no systematic, evidence-based educational program for PFA has been established in the field of mental health in the country. Considering an increasing recognition of the need for PFA training for all personnel who potentially work with disaster survivors, providing an empirically supported educational program is crucial.

The present study aimed to examine PFA training effects in nonprofessional and professional health care providers in Korea. Based on the RAPID-PFA program [13], the PFA field operation guide of the NCTSN [4], and the WHO PFA manual [3], a modified version of PFA training was developed for the study. The program included a didactic portion of the basic theory of disaster mental health in addition to common educational contents of PFA such as core activities, behavioral guidelines, and self-care. The content of our didactic lecture is similar to the existing PFA programs noted above, but we modified the simulation practice part of the program in order to facilitate feedback-based learning. First, we developed two simulations (earthquake and fire) with 20 case scenarios for each, in which roles of the cases were specified (see S1 File). Secondly, we developed behavioral checklists to facilitate self-evaluation and interactive feedback regarding general and specific behavioral guidelines tailored to the cases (see S2 File). This simulation program is structured using standardized instructions and time limit for each segment of the practice. The simulation program was developed by the third and fourth authors of the study with the consultation of eight disaster mental health experts in Korea (4 clinical psychologists, 1 psychiatrist, and 3 nurses). An overview of the PFA training used for the study follows.

### Overview of PFA training

A PFA training program with didactic and simulation-based methods was developed for health care professionals, paraprofessionals, and nonprofessional personnel in Korea. The program was delivered as a one-day workshop with a 3-hour didactic lecture and 3-hour simulation-based practice. The goal of the didactic lecture was to increase knowledge about disaster mental health and PFA. Contents of the lecture covered the following domains: 1) theoretical overview of disaster mental health including common responses of disaster survivors, 2) introduction to PFA and basic principles, 3) general behavioral guidelines when working with disaster survivors, 4) core activities of PFA, and 5) self-care. Basic principles of PFA were based on Hobfoll et al.’s [10] recommendations. Behavioral guidelines included attitudes and specific behaviors that should be avoided as well as recommended courses of actions when working with various populations (i.e., children and adolescents, older adults, people with disabilities). Core activities of PFA included the following: 1) initial contact and rapport building, 2) providing support and safety, 3) stabilization, 4) information gathering for immediate needs, 5) problem solving, 6) making connections to social support systems, 7) providing information regarding adaptive and maladaptive coping, and 8) linkage to community institutions for health care services. The didactic lecture for self-care included common stress responses of helpers, cognitions related to burnout, individual strategies for self-care, and institutional guidelines for protecting helpers from burnout. Finally, ethical guidelines for individuals working with disaster survivors were presented.

The goal of the 3-hour simulation-based practice was to increase PFA skills and competency in working with disaster survivors. The recommended number of group members for this practice training was 20 to 30, although it was designed for a maximum of 40 people in a group. Two scenarios were developed for simulation-based practice training. One scenario was about a situation following an earthquake (natural disaster), and the other was about a situation following a fire in a building (man-made disaster). For each scenario, brief descriptions for 20 survivors were developed. These survivor scenarios included people with various emotional and behavioral responses and diverse populations such as children, adolescents, adults, older adults, pregnant women, and people with disabilities. For self-evaluation and interactive feedback about role-play performance, behavioral checklists were developed. These checklists had three forms: 1) common checklists for general behavioral guidelines of PFA, 2) individual case checklists for specific, adaptive responses tailored to each survivor case, and 3) team-building checklists for helper groups. Example questions for common checklists about general behavioral guidelines of PFA included “When meeting the survivor for the first time, did you introduce your name, affiliation, role, and purpose of the visit?” “Did you ask permission to speak with them?” “Did you ask whether they needed anything?” or “Did you provide help that would comfort a suffering person (e.g., providing information at the scene related to the rescue, tissues, water, or drinks when needed)?” Example questions for specific, adaptive responses tailored to each survivor case included questions checking whether the helper properly behaved in accordance with survivor needs. For example, for a survivor case that reported flashbacks, specific behavioral checklist questions included “Did you say that it is a possible response to traumatic experience?” “Did you teach stabilization techniques to help him reduce fear?” “Did you let him know that if the flashback continues, he needs professional help” “If needed, did you refer him to mental health institutions?” All participants were instructed to respond “yes” or “no.”

The 3-hour practice started with an overview of basic communication skills. Then the group was divided into two subgroups for role-play. One group played survivor roles as a team, and another group played helper roles at the first simulation practice, and then, for the second simulation, they switched their roles. For each simulation, 10 minutes were given for preparation and another 10 minutes were given for role-play. After finishing the first role-play, behavioral checklists for general behavioral guidelines were distributed to all participants. All participants rated the behavioral checklists for their own behaviors and/or behaviors of people they interacted with during the role-play. Group discussions followed, and providing interactive feedback was strongly encouraged for all participants. After completing the second role-play, behavioral checklists for specific, adaptive responses tailored to each survivor case in addition to checklists for general behavioral guidelines were given to the participants. A small group discussion was conducted to facilitate interactive feedback about their performance, and then all groups shared their feedback and comments. This simulation practice was led by two facilitators: one main group leader and a co-leader.

## Study 1

We sought to examine whether there would be significant training effects of a PFA program on knowledge and skills. In doing this, we conducted a study with undergraduates who were studying psychology for their BA degree and graduate students studying clinical psychology for their MA degree. We expected that there would be a significant increase in PFA knowledge and perceived competence in PFA skills after participating in a one-day workshop of the PFA program.

## Materials and methods

### Participants and procedure

Thirty-seven undergraduate and graduate students in the department of psychology voluntarily participated in a one-day workshop held on August 30, 2016 at Keimyung University. On arrival, they received an information sheet about the study and completed a consent form. All participants completed a 3-hour didactic lecture and a 3-hour simulation-based practice. At the end of the workshop, a small gift was given for their participation. The study was approved by the Institutional Review Board of Keimyung University (40525-201506-HR-41) in Korea.

### Program deliverers

The didactic lecture was delivered by a licensed clinical psychologist. The simulation-based practice was delivered by a group leader and a co-leader, who were licensed clinical psychologists, and the observers were graduate students majoring in clinical psychology.

### Measures

#### PFA Knowledge

PFA knowledge was assessed using a set of two equivalent tests with 10 items each. Initially, 47 preliminary test items were developed by two doctoral-level clinical psychologists and five graduate students (1 currently enrolled in a doctoral program and 4 in a master’s degree program) to cover the content of the didactic part of the PFA training. Fifteen mental health professionals rated the degree of suitability of preliminary test items on a 5-point Likert scale (0 = not at all, 4 = considerably). Twenty items scoring above 3 on average in the degree of suitability were selected. These items were then administered to 38 mental health professionals (5 psychiatrists, 5 clinical psychologists, 1 social worker, 1 nurse, and 26 graduate students) to assess the level of item difficulty. Considering item difficulty and subareas, two parallel forms of the scale with 10 items each were chosen to use for the study. Both forms included questions with a similar level of difficulty covering five domains (theoretical overview of disaster mental health, overview of PFA and basic principles, general behavioral guidelines, core activities of PFA, and self-care), as mentioned in the previous section. Pre- and posttest questions for PFA knowledge in English and Korean are provided (see S3 File).

#### Perceived competence in PFA skill

Perceived competence in PFA skill was assessed using the Disaster Mental Health Competency Scale [18]. The scale was developed to assess the overall competency of disaster mental health professionals. In this study, nine items assessing perceived competence in PFA skills were used to examine the training effect of the simulation-based practice component of PFA training. These nine items included 3 items assessing problem solving (e.g., “I can analyze problems occurring at the disaster scene and find solutions”), 3 items assessing communication skills (e.g., “I am able to understand the demands of the survivor in a disaster situation”), and 3 items assessing ability to convey information (e.g., “I can convey information that is of actual value to the survivor in a disaster situation”). The internal consistency coefficient (Cronbach’s α) for the nine items was .94 for the pretest and .91 for the posttest.

#### Statistical analysis

Prior to statistical analyses, the normality of the data for each variable was examined. Skewness ranged from -0.08 to 0.86, and kurtosis ranged from -0.36 to -0.80. P-P plots and Shapiro-Wilk test displayed a normal distribution. As such, paired samples t-tests of change over time were performed for PFA knowledge and perceived competence in PFA skill, respectively. Paired sample t-tests of change over time required multiple tests; Bonferroni correction was applied to protect against inflated Type 1 error rates. As such, only those with p values of < .025 were considered significant. As 51.4% (n = 19/37) of the participants had at least one disaster related lecture or training, we then conducted further sensitivity analysis using a repeated measures ANOVA, with Group (those without a prior lecture vs. those with a prior lecture) as the between-subjects factor and Time (pretest vs. posttest) as the within-subjects factor. We coded those with prior lecture as 1 and those without as 0. We aimed to explore whether prior training plays a moderator role.

## Results

### Participant characteristics

Age of the participants ranged from 18 to 36 years old (M = 24.83, SD = 3.75); 63.2% (n = 24) of them were women and 34.2% (n = 13) were men. Of the participants, 23.7% (n = 9) had completed high school, 52.6% (n = 20) had a university degree, and 21.1% (n = 8) held a postgraduate degree. Most of them were single (n = 35), and 5.4% (n = 2) were married. Fifty percent (n = 19) had previously completed at least one disaster or PTSD-related education program (e.g., Psychological First Aid). Participant characteristics are presented in Table 1.

**Table 1:** Characteristics of the student sample (N = 37).

**Age, M (SD)**	24.83 (3.75)	
**Gender**		
**Men**	35.1	13
**Women**	64.9	24
**Education level**		
**Undergraduate students**	24.3	9
**Graduate students**	75.7	28
**Marital Status**		
**Single**	94.6	35
**Married**	5.4	2

### Effect of training program on each measure

Paired samples t-tests confirmed significant increases from pre- to posttest in PFA knowledge, t (36) = -8.04 (mean change = +2.38), p < .001, d = 1.42, and perceived competence in PFA skill, t (36) = -9.74 (mean change = +10.70), p < .001, d = 1.61 (see Table 2). We then conducted further sensitivity analysis to investigate whether prior training would be a moderator. Using a repeated measures ANOVA, with Group (those without a prior lecture vs. those with a prior lecture) as the between-subjects factor and Time (pretest vs. posttest) as the within-subjects factor, we found a significant main effect of Time, F (1, 35) = 62.78, p < .001, ηp2 = .64, with more increased knowledge at posttest than at pretest (5.19 vs. 7.57). A main effect of Group, F (1, 35) = 5.31, p = .03, ηp2 = .13, was also significant, indicating that those who took prior training, related to disaster relative to those without, reported higher scores on knowledge (5.63 vs. 4.72 at pretest, 8.10 vs. 7.00 at posttest). However, the Group x Time interaction, F (1, 35) = .10, p = .74, was not significant. For perceived competence in PFA skill, a significant main effect of Time, F (1, 35) = 95.67, p < .001, ηp2 = .73, was found, with more increased perceived competence in PFA skill for the posttest than the pretest (12.54 vs. 23.24). However, there was no significant main effect of Group, F (1, 35) = .07, p = .80, nor any significant interaction effect between Group and Time, F (1, 35) = 1.11, p = .30. All data underlying the findings in Study 1 are fully available without restriction (see S1 Data).

**Table 2:** Pre- and posttest changes on each measure of the student sample (N = 37).

	Pretest	Posttest			
	Mean(SD)	Mean(SD)	*t*	*p*	*d*
**PFA Knowledge**	5.19(1.70)	7.57(1.64)	-8.04	< .001	1.42
**Perceived competence in PFA skill**	12.54(6.28)	23.24(5.85)	-9.74	< .001	1.61

## Study 2

In Study 1, we found that PFA training with didactic and simulation-based practice yielded large effect sizes in increasing PFA knowledge and perceived competence in PFA skill in a student sample. However, further questions remained, such as would the PFA program used in the present study be still effective when applied to mental health professionals? According to Everly et al. [13], the increased perception regarding one’s capacity related to PFA functions as a resilience factor for disaster mental health providers. In this context, we investigated whether one’s perceived preparedness and confidence to provide PFA for future disasters would be altered as a result of PFA training. Another important factor that should be considered is the increased willingness to offer PFA to survivors in the context of a disaster, which might lead to the availability of more PFA providers. As such, in Study 2, we hypothesized that the PFA training in the present study would affect one’s perception of willingness, preparedness, and confidence to provide psychological assistance (e.g., psychological first aid), along with increased PFA knowledge and perceived competence in PFA skill for mental health practitioners.

## Materials and methods

### Participants and procedure

A total of 82 school counselors from two cities in Gyeongbuk province in South Korea participated in the study. Out of 82 participants, 6 participants did not complete either the pretest or posttest, which was handled as missing data using multiple imputation. Nine participants were excluded from the final analysis as they did not correctly report their tracking numbers; therefore, their pretests and posttests were not correctly matched for the analysis. Finally, 73 participants were included in the final analysis. Their demographic information is presented in Table 3.

**Table 3:** Characteristics of the school counselor sample (N = 73).

Characteristics of the sample	%	n
**Age, M (SD)**	41.46 (9.86)	72
**Gender**		
**Men**	5.5	4
**Women**	93.2	68
**No response**	1.4	1
**Education level**		
**High school**	2.7	2
**College/university**	49.3	36
**Graduate school**	39.7	29
**No response**	8.2	6
**Duration of employment**		
**< 1 year**	4.1	3
**1 to < 3 years**	30.1	22
**3 to < 5 years**	27.4	20
**5 to < 10 years**	20.5	15
**More than 10 years**	9.6	7
**No response**	8.2	6

*Note*. One person did not report age information.

After an earthquake (magnitude 5.8), the strongest one ever in the country, occurred in September 12, 2016 in Gyeongbuk province in South Korea, two one-day PFA workshops were provided to school counselors in Gyeongbuk province to prepare for upcoming disasters. The workshops were held on December 21 in Gyeongju and December 22, 2016 in Gimcheon. Both workshops were supported by the Office of Education in Gyeongbuk province. All participants were informed about the study prior to the didactic lecture, and pre- and post-assessments were administered to those who signed the consent form. On arrival, they were given a small booklet including the workshop schedule and the content of the didactic lecture. They then had the 3-hour didactic lecture, followed by a lunch break and simulation-based practices. Participants were randomly allocated to one of two groups for the simulation-based practice. Random assignment was conducted using a colored sticker on a booklet (red or green). After the didactic lecture, they were informed that they needed to check which color sticker they had, and then they went to Room A or Room B accordingly. Pre- and post-assessments were conducted before the didactic lecture and immediately after simulation-based practice, respectively. The study was approved by the Institutional Review Board of Chungbuk National University (CBNU-201611-BMSB-385) in South Korea.

### Program deliverers

The didactic lectures were delivered by a licensed clinical psychologist. Simulation-based practices were led by a group leader and a co-leader. The group leader and co-leader were licensed clinical psychologists.

### Measures

#### PFA knowledge

The PFA knowledge questionnaire was the same as the one used in Study 1.

#### Perceived competence in PFA skill

The perceived competence in PFA skill questionnaire was the same one used in Study 1.

#### Perceived willingness, preparedness, and confidence to provide psychological assistance for future disasters

Perceived willingness, preparedness, and confidence to provide psychological assistance (e.g., psychological first aid) to survivors for future disasters were assessed using the following questions: “If any of the following disasters were to occur in the community you live, would you be willing to provide (willingness), would you be prepared to provide (preparedness), and would you be confident or competent (confidence) in providing psychological assistance (e.g., psychological first aid) to survivors?” Responders rated the degree of willingness, preparedness, and confidence from 0 (not at all) to 4 (considerably) for four types of disasters: earthquake, fire, typhoon, and explosion. The questionnaire we used for this study is provided (see S4 File). The possible score range for each of the scales (i.e., willingness, preparedness, and confidence) was 0 to 16. The internal consistency coefficients (Cronbach’s α) for each scale were as follows: .92 at pretest and .95 at posttest for willingness, .95 at pretest and .95 at posttest for preparedness, and .97 at pretest and .96 at posttest for confidence.

#### Missing data and statistical analysis

Missing data at pre- and posttest were handled with multiple imputation using SPSS version 22. Although performing five to ten imputations is sufficient [19], Graham, Olchowski, and Gilreath [20] recommended more imputations than the number usually considered. Thus, we generated 50 imputed data sets. Paired samples *t* tests were conducted by calculating average mean differences with adjusted standard error from 50 imputed data sets. Degree of freedom was obtained based on the formula suggested by Barnard and Rubin [21].

The normality of the data was examined using skewness, kurtosis, P-P plot, and Shapiro-Wilk test. Shapiro-Wilk test indicated that the data for PFA knowledge, perceived preparedness, perceived confidence, and perceived willingness except for perceived competence in PFA skill appeared to be non-normal. However, skewness ranged from -0.12 to 0.62, and kurtosis ranged from 0.19 to 1.21. P-P plots showed that all variables roughly followed a normal distribution. Furthermore, a small deviation from normal distribution when the sample size is larger than 50 rarely influences results of parametric test [22]. Accordingly, paired samples t-tests of change over time (parametric test) were performed. The Bonferroni correction was applied as multiple paired samples t-tests were performed (significant p value < .0125).

## Results

### Participant characteristics

The demographic characteristics of the participants are presented in Table 3. Age of the participants ranged from 24 to 64 years old (M = 41.46, SD = 9.86): 93.2% (n = 68/73) were women and 5.5% (n = 4/73) were men. A total of 49.3% (n = 36/73) had received a university degree, 39.7% (n = 29/73) had a graduate degree, and 2.7% (n = 2/73) completed high school. Of the participants, 45.2% (n = 33/73) were school counselors; 21.9% (n = 16/73) were psychologists; 1.4% (n = 1/73) were social workers; 21.9% (n = 16/73) checked the box for “non-specified,” and 9.6% (n = 7/73) did not report their job. Most participants (91.8%; n = 67/73) had completed at least one disaster-related training (e.g., psychological first aid).

### Effect of training program on each measure

As shown in Table 4, the increases in PFA knowledge, t (68.073) = -5.29 (mean change = +1.21), p < .001, d = .63, and perceived competence in PFA skill, t (69.803) = -9.87 (mean change = +6.62), p < .001, d = 1.10, were significant. The predicted differences from pre- to posttest in perceived preparedness, t (62.968) = -8.33 (mean change = +0.93), p < .001, d = 1.05, and perceived confidence, t (59.675) = -5.98, p < .001 (mean change = +0.60), d = 0.64, were also significant. Perceived willingness failed to reach statistical significance, t (65.588) = -0.75, p = 0.46 (mean change = -.07).

**Table 4:** Pre- and posttest changes on each measure of the school counselor sample (N = 73).

	Pretest	Posttest			
	Mean (SD)	Mean (SD)	*t*	*p*	*d*
**PFA Knowledge**	4.74 (2.01)	5.90 (1.84)	-5.29	< .001	0.63
**Perceived competence in PFA skill**	17.23 (6.57)	23.78 (5.35)	-9.89	< .001	1.10
**Perceived willingness**	2.60 (0.92)	2.67 (0.76)	-0.75	0.46	-
**Perceived preparedness**	1.43 (0.95)	2.38 (0.82)	-8.33	< .001	1.05
**Perceived confidence**	1.74 (1.00)	2.34 (0.86)	-5.98	< .001	0.64

*Note*. Missing data at pre- and posttest were handled with multiple imputation.

As participants practiced only two types of disaster scenarios (earthquake and fire) in the mock simulations but were asked to rate the level of their perceived willingness regarding four types of disasters (earthquake, fire, typhoon, and explosion), additional analyses were conducted to check whether participants reported their perceived willingness to offer PFA differently across four types of disasters. Separate paired samples t-tests of change over time using two types of combined disasters (earthquake and fire or typhoon and explosion) were performed, and no significant results were found for earthquake and fire, t (66.722) = - 0.87, p = .39 and for typhoon and explosion, t (66.373) = - 0.60, p = .55. All data underlying the findings in Study 2 are fully available without restriction (see S2 and S3 Data).

## Discussion

One of the big challenges of the public mental health system in disaster contexts is that there is a serious shortage of qualified practitioners to offer PFA. Additionally, there is a lack of studies investigating the effectiveness of the PFA training program. Training a qualified disaster mental health provider with an evidence-based PFA program could lead to a well-organized community response following a disaster. In this sense, the present study aimed to explore the effectiveness of a one-day PFA program using a series of assessments, such as PFA knowledge, perceived competence in PFA skill, perceived willingness, preparedness, and confidence to offer psychological assistance. The main findings were as follows: a one-day PFA training consisting of a didactic lecture and a simulation-based practice yielded medium to large effect sizes. Perceived willingness failed to reach significance.

The results of the present study using the 6-hour PFA program are consistent with Everly et al. [13]’s study using the RAPID-PFA program. More specifically, we found a medium-sized effect on PFA knowledge and perceived confidence to provide psychological assistance for future disasters, and large effects on perceived competence in PFA skills and perceived preparedness. Similarly, Everly et al. [13] reported a small to medium effect on knowledge and confidence in personal resilience (attitudes), with a large effect on self-efficacy in application of interventions (perceived skills). These results indicate that a brief 6-hour PFA program might be sufficient to increase perceived competence in PFA skills for disaster mental health providers. However, the results show that there was a smaller effect size on perceived confidence relative to skills, suggesting that we need to take the program a step further to foster perceived confidence, a resilience factor as noted by Everly et al. [13]. What caused a small to medium effect size in perceived confidence? Presumably, a short-term one-day workshop might not be enough to alter participants’ perception of confidence to function in a disaster context. If this is the case, a prolonged PFA training program might be helpful to foster one’s perception of capability to offer PFA. Another possibility is that other components might affect instillation of perceived confidence. The program we conducted is a virtual-based, not reality-based. Participants’ motivation or the level of involvement in a given simulation might not be sufficient for believing that they have enough confidence to function in an actual disaster. A future study is warranted to evaluate whether repeated practices with mock simulations more like actual disasters would produce larger effect sizes. Follow-up studies to examine if trainees use the PFA program in a real-world disaster, and the extent to which there is a change in perceived confidence, might be another possibility.

On the other hand, consistent with Everly et al.’s [13] study, PFA knowledge was not as greatly improved as we originally expected. We presume that this might be due to assessment points. As done in Everly et al. [13], we asked participants to complete all the assessments before the didactic lecture and immediately after simulation practice training. The effect of training on knowledge through the didactic lecture in the morning session might be overridden by effects of simulation practices conducted in the afternoon session; that is, knowledge they learned might be overshadowed by the simulation practice. According to Ebbinghaus’ forgetting curve, without a retention period a rapid initial loss of memory occurs [23]. Given that professional knowledge related to disasters and PFA is a critical factor for disaster mental health providers’ decision making to take helpful and unhelpful actions in disaster contexts, more endeavors to examine strategies to foster PFA knowledge are worthwhile for a future study.

Even though participants reported that their PFA knowledge, perceived competence in PFA skill, and perceived preparedness were improved to offer PFA through a one-day workshop, their willingness to offer PFA when facing a real disaster situation did not improve. Why did the change in perceived willingness to offer PFA training not reach significance? As there was not a debriefing session for this study, we were not able to analyze the reasons underlying this result. However, we presume that the insufficient increase in knowledge and perceived confidence shown by the smaller than expected effect size for a one-day 6-hour training might influence the non-significance. Another possibility that participants may have reported their perceived willingness to offer PFA differently across 4 types of disasters was examined, but this hypothesis was not supported as reported in the result section. As such, this possibility was excluded.

Other consideration regarding why perceived willingness to provide PFA did not change in Study 2 was the higher baseline level of perceived willingness among the school counselors. The question of perceived willingness to deliver PFA was measured using a 5-point Likert scale (0 = not at all, 1 = disagree, 2 = medium, 3 = agree, 4 = considerably). The pretest mean for the perceived willingness of the school counselors was 2.61, which falls into medium to agree on average. Compared to the baseline means for perceived preparedness (pretest mean = 1.43) and perceived confidence (pretest mean = 1.74), the mean of perceived willingness was above average at the baseline. This indicates that the school counselors participating in Study 2 had decent motivation to provide psychological assistance even before they received the PFA training. This might have contributed to the nonsignificant changes in willingness to participate in PFA after training. Regretfully, we failed to examine this in the student sample in Study 1. Given that PFA training is offered to nonprofessional volunteers or paraprofessionals, and that the increased willingness to offer PFA to survivors in a real-world disaster emergency might be a critical factor that affects an individual’s preparedness for a more systematic and skillful response, including content in PFA training designed to increase the perceived willingness to offer PFA is warranted as a future direction in this area.

Finally, we conducted a sensitivity analysis in Study 1 to examine whether prior training experience would influence the effect of training in the present study. At both time points (pretest and posttest), those with prior training reported slightly more knowledge related to disaster compared to those without. However, the hypothesis that prior training would yield significant differences in PFA knowledge and perceived competence in PFA skill before and after the training was not confirmed by our data.

Developing and disseminating evidence-based PFA programs are essential. Despite considerable efforts by WHO, NCTSN, and many international scholars and practitioners, the current status of scientific evidence for PFA is evidence-informed, rather than empirically supported. Expert consensus and practical experiences are important ingredients in the development of evidence-based programs. Yet, more evidence for training effects is needed. Another important issue is how to disseminate the program. The organization of the NCTSN provides a good model, in which a collaborative network was formed among the headquarters center, academic and medical-center affiliated treatment centers, and community treatment and service centers [24]. Furthermore, development of diverse means of disseminating PFA training programs is needed. In addition to the format of a one-day workshop, other training methods, including online training and simulation training using live or video demonstrations, need to be developed and tested for effectiveness.

There are several limitations that should be noted. Firstly, this study did not include a controlled counterpart (no training control condition), which limits the ability to check for a genuine effect of the PFA training program. For practical and ethical reasons, we were not able to include a no training condition, as all the participants wanted to complete a one-day workshop because they needed to be able to immediately offer PFA in their communities. As previously mentioned, this training program was prioritized in two sites that had earthquakes 3 months prior to the workshop. Nonetheless, a randomized controlled study including a controlled counterpart is considerably important for a next step to test for a genuine training effect. Furthermore, a future study comparing PFA training effects with and without simulation will be necessary. Secondly, although we provided reliability using internal consistency coefficients, the newly developed competency measures warrant further psychometric examination.

Despite the limitations noted above, the present study makes an important contribution to the field. Most of all, the study provides an empirically supported PFA training program to nonprofessional and professional health care providers in Korea. The strengths of the PFA program included the use of specific case scenarios and behavioral checklists. Behavioral checklists were used as a tool to provide interactive feedback and to increase self-perception about participants’ own behaviors. In addition, the simulation practice is firmly structured such as providing specific time frames and tasks for each segment of role-play. The study provides preliminary evidence indicating that the combined method of didactic and experiential learning could enhance knowledge and perceived competence in skills for providing PFA, as well as perceived preparedness and confidence in future psychological assistance in the context of disasters. Further research using outcome measures including other’s rating scales, in addition to self-rating scales, to support this conclusion is warranted.

## Supporting information

S1 Data Dataset of Study 1. (SAV)

S2 Data Dataset of Study 2. (SAV)

S3 Data Imputed dataset of Study 2. (SAV)

S1 File Simulations and case scenarios. (PDF)

S2 File Behavioral checklists. (PDF)

S3 File PFA knowledge tests. (PDF)

S4 File Perceived willingness, preparedness, and confidence. (PDF)
