# Supplementary material for: A preliminary evaluation of the training effects of a didactic and simulation-based psychological first aid program in students and school counselors in South Korea
Source: PLoS One. 2017 Jul 17;12(7):e0181271. doi: 10.1371/journal.pone.0181271 (PMC5513559; doi:10.1371/journal.pone.0181271)
Supplement: S1 File — (PDF) [file pone.0181271.s004.pdf]

Supporting Information S1
Simulations and Case Scenarios

SIMULATION 1: Earthquake Scenario

A magnitude 6 earthquake occurs in region 00 resulting in 300 deaths, and residents in the vicinity have all gathered at a shelter as directed by an announcement. Less than a day after the earthquake, the atmosphere is still chaotic. A portion of the people gathered at the shelter are searching to see whether their families have arrived safely and, every time a new person arrives those who have not been able to reunite with their family, check to see if it is someone they recognize. Some people attempt to leave the shelter saying they are going to find their family, but they are restrained due to the dangers presented by aftershocks.

Carefully examine the roles below and choose a role to adopt. Set up the role of the individual in the disaster and during the simulation preparation time (approximately 10 minutes), think about how to perform the role you have chosen. Please participate earnestly during the activity. In a moment, all of you will arrive at the rest area that has been prepared in the disaster situation. There is no need to become fixed on the role, you may respond naturally to the psychological worker’s treatment.

Survivor Cases

NO.	Main problem	Content
1	Child	“A” is a 10-year-old girl. A’s mother is trying to comfort her, but A does not stop crying.
2	Guardian	Ms. B (30 years old) is the mother of A. She is doing her best to comfort her daughter, but the daughter will not stop crying. The mother is having a difficult time knowing what to do given the chaotic circumstance.
3	Guilt	Ms. C is a woman in her late 40s. She was rescued and taken to the shelter. She is not answering any questions about her current state, and suddenly begins to cry as she recounts feeling sorry for escaping the scene of the earthquake while ignoring the cries of others.
4	Assistant	Mr. D is a high school student. He was rescued and has gathered with some friends at the shelter. Mr. D performed a headcount of people in the shelter, checked on his injured friends, and is showing leadership by taking care of his friends.
5	Hyperarousal, youth	Mr. E is a high school student. He was with Mr. D when the earthquake happened. Mr. E is startled at the sound of any object falling, and goes out of his way to avoid anyone passing by him. He is curled up with his arms wrapped around himself.

6	Fear, youth	Ms. F is a high school student. When the building collapsed, she was with Mr. D and Mr. E, and was rescued and taken to the shelter with no major injuries. When she arrives at the shelter, she continues to want to be left alone and does not move from the corner.
7	Elderly	Mr. G is in his 70s. He was safely rescued from the scene of the earthquake. When the ambulance worker asks basic questions about his condition, Mr. G refuses help and tells the worker to "go away."
8	Anger	Ms. H is in her 20s. Her parents are missing due to the earthquake. When a crew of reporters comes to cover the situation, she yells, "Don't take my picture!"
9	Service grievance	Mr. I is in his 30s. His wife is missing and he is staying at the shelter. He is complaining to the administrative staff about the dilapidated state of the shelter.
10	Child	J is the 7-year-old child of Ms. K. J is startled by the earthquake and will not stop asking his mother questions. His mother is not responding to his questions so J wanders around asking what has happened. His mother scolds him excessively for this behavior.
11	Guardian, stress	Ms. K is in her 30s. She appears to be irritated as she yells at her child who looks to be of preschool age, telling him to sit still.
12	Anger	Mr. L is in his 30s. Mr. L says his family still has not arrived at the shelter as he puts on gear and says that he is going out to find them. When some officials attempt to stop him in front of the door, he yells, "What can you do?"
13	Victim mentality, paranoid	Mr. M is in his 50s. He says he is not feeling well and demands help. When the psychological personnel ask for Mr. M's personal information, he responds harshly asking what they intend to do with it. He has an excessively defensive attitude to filling out any personal information.
14	Service grievance	Ms. N is in her 40s. She is demanding her seat be changed, saying that the person next to her is too loud.
15	Service grievance	Mr. O is in his 80s. He is holding a brochure from the headquarters and is yelling. He complains the letters on the brochure are too small.

16	Dazed	Ms. P is in her 50s. She has entered the shelter where current missing families and families of the deceased are staying. She is not eating or sleeping, and waits in a dazed state for her daughter to be rescued.
17	Anger	Mr. Q is in his 50s. He lost his wife when a building collapsed during the earthquake. He is staying in the shelter and yelling to others that, “The shelter could collapse, too!” He is inciting other people to move to other buildings.
18	Assistant	Mr. R is in his 40s. He was rescued quickly from the scene of the earthquake, and did not sustain any serious injuries. He calmly assesses which other people are at the disaster scene and is ready to talk about how others are doing. He is bringing water to others and helping proactively.
19	Elderly	Mr. S is in his 70s. He was rescued quickly from the scene of the earthquake and is transported to a safe location without sustaining any serious injuries. He is asked for some basic personal information, but appears to have a hard time understanding.
20	Service grievance	Ms. T is in her 30s. She was rescued with her husband from the scene of the earthquake. She says her husband was seriously injured and taken to a nearby hospital. However, she says she does not trust local hospitals and is yelling that he must be taken to a university hospital.

SIMULATION 2 Fire Scenario

At 8 AM, a fire started on the 8th floor of an apartment building. The fire spread to other floors until the entire upper portion of the building was engulfed in flames. The scale of the fire grew as it moved to an adjacent apartment building. The fire fighters who responded were able to extinguish the flames easily, but toxic gas emitted from the building materials resulted in the death of 200 people. Survivors who instantly lost family members received medical treatment and are living in a temporary shelter that has been prepared in the auditorium of an elementary school.

Carefully examine the roles below, and choose a role to adopt. Set up the role of the individual in the disaster and, during the simulation preparation time (approximately 10 minutes), think about how to perform the role you have chosen. Please participate earnestly during the practice. In a moment, a few people will arrive to help you. There is no need to become fixed on the role, and you may respond naturally to the psychological worker’s treatment.

Survivor Cases

NO.	Main problem	Content
1	Anger	Mr. A is in his 30s. Mr. A says his wife died from toxic gas. He is yelling while asking where the construction company is and demands punishment for those responsible.
2	Dazed State	B is a middle school student. While living at the temporary shelter he is unable to focus and does not respond when people speak to him.
3	Flashback sleep disorder	Mr. C is in his 30s. He is in a somewhat stable condition after being rescued, but reports that he keeps thinking about images of his house of fire and the seriously injured people being rescued.
4	Helper	Ms. D is in her 50s. During the week that Ms. D has been living at the temporary shelter, she has been speaking to survivors who are alone and bringing water and blankets for others.
5	Elderly, fear	Ms. E is in her 70s. Ms. E has nightmares of the fire and is unable to sleep. While looking for a fire extinguisher, she says the shelter might also catch on fire. She demands that the shelter provide her with a spot near the exit.
6	Service grievance	Mr. F is in his 50s. He says he does not feel well and asks whether he can receive free treatment at the hospital. The psychological personnel direct him to a place where he can receive free treatment, but Mr. F says he does not like that hospital and demands a different hospital.

7	Anger Paranoia symptoms	Mr. G is in his 30s. Mr. G claims the shelter's psychological personnel are mocking him and not giving him enough food while throwing his lunch at the psychological personnel.
8, 9	Stress, youth	H and I are middle-school students. While joking with each other they suddenly start fighting, swearing at one another and telling each other to watch their mouths.
10	Fear	Ms. J is in her 20s. While crying, Ms. J says she is afraid to be alone and asks the psychological personnel for help.
11	Pregnant mother	Ms. K is in her 30s and about 3 months pregnant. At the shelter, Ms. K cries and complains of a stomachache.
12	Guardian	Ms. K's husband Mr. L (in his 30s) is angry with the psychological personnel saying that there is no information or treatment for pregnant women and that putting everyone in the same place is unfair.
13	Visually impaired, fear	Mr. M is in his 80s and is visually impaired. He is currently living alone at the shelter. He has a frightened expression at the chaotic noise of other victims moving different items around. He gropes the floor and attempts to stand up.
14	Service grievance	Mr. N is in his 40s. Mr. N is yelling that he has not received any assistance since he arrived, and is taking water, food, blankets, and other items as he pleases. The psychological worker explains that these items will be distributed in order, but Mr. N obstinately elbows the psychological worker out of the way and takes the items.
15	Child with no guardian	O is an 8-year-old boy. He was rescued from the building and brought to the shelter. O is crying because he cannot find his parents.
16	Helper	Mr. P is in his 30s. While living at the temporary shelter he helps the psychological personnel and comforts other survivors.

17	Anger	Ms. Q is in her 50s. Ms. Q says that her family still has not arrived and attempts to leave saying that she is going to find them herself.
18	Dazed state	Mr. R is in his 20s. He sits in a corner of the temporary shelter without speaking. He does not respond to questions or requests from others at the shelter.
19	Guardian stress	Mr. R's mother Ms. S (in her 40s) is talking about her grievances and says her son has not spoken all day. She says the situation is making her more sensitive and frustrated.
20	Hyperarousal	Mr. T is in his 60s. When the psychological worker approaches to ask about some personal information Mr. T yells at him to "Go away!"

Simulations and Case Scenarios (In Korean)

SIMULATION 1 지진 시나리오

00지역에서 규모 6 지진이 발생하여 300여명의 사상자가 발생하였고, 인근 주민들은 모두 안내방송에 따라 대피소로 모여들었습니다. 지진 발생 후 하루가 채 지나지 않아 아직까지는 어수선한 분위기입니다. 대피소에 모여든 사람들 중 일부는 자신의 가족이 안전하게 도착했는지 찾아 다니고 있고, 아직 가족을 만나지 못한 사람들은 새로운 사람들이 도착할 때마다 자신의 가족인지 확인하고 있습니다. 일부 사람들은 가족을 찾아야겠다며 밖으로 나가려고 하지만 여진의 위험이 있어 제지 당하고 있습니다.

아래의 역할을 천천히 살펴보시고, 각자가 맡을 역할을 선택해주십시오. 재난 피해 상황에 있는 대상자 역할을 설정하고, 시뮬레이션 준비시간 동안(약 10분) 선택한 역할을 어떻게 연기하면 좋을지 생각해 주시면 됩니다. 연습 동안 진지하게 참여해 주십시오. 잠시 후 여러 분은 재난 상황에서 마련된 쉼터에 도착하게 될 것입니다. 역할에 고착될 필요가 없고, 심리요원의 대처에 따라 자유롭게 반응하시면 됩니다.

생존자 사례

번호	주요문제	내용
1	아동	A양은 10살 여자아이이다. A양의 어머니가 계속해서 아이를 달래고 있지만 A양은 울음을 그치지 않는다.
2	보호자	B씨는 A양의 어머니이다(30대). 딸을 달래려 최선을 다하고 있지만 아이가 울음을 그치지 않고 있다. B씨는 본인도 혼란스러운 상황에서 아이에게 어떻게 대해야 할지 몰라 힘들어 하는 상황이다.
3	죄책감	C씨는 40대 후반 여성이다. 무사히 구조되어 대피소에 도착했다. 자신의 상태에 대해서 묻는 질문에 답하지 않고, 다른 사람들이 소리치는 것을 무시한 채 혼자 지진 현장을 벗어난 것이 미안하다며 갑자기 울음을 터트린다.
4	조력자 청소년	D군은 고등학생이다. 무사히 구조되어 대피소에 친구들과 같이 모여 있다. D군은 인원수를 파악하고, 다친 친구들을 확인하는 등 리더십 있는 모습으로 친구들을 챙기고 있다.
5	과각성 청소년	E군은 고등학생이다. D군과 함께 있다가 지진을 경험하였다. 물체가 떨어지는 소리에 화들짝 놀라고, 누군가가 자신의 곁을 스쳐지나가기만 해도 과도하게 몸을 피하며 팔로 몸을 감싸고 웅크리는 자세를 취한다.

6	두려움<br>청소년	F양은 여자 고등학생이다. 건물이 붕괴 될 때 D군, E군과 함께 있었으며, 큰 상처 없이 구조되어 대피소로 후송되었다. 대피소에서 도착해서는 계속 혼자 있고 싶어 하며, 구석에서 움직이지 않으려고 한다.
7	노인	G씨는 70대 노인이다. 지진 현장에서 무사히 구조되었다. 구급 대원이 기본 신상을 물으려고 하자 '저리 가라'며 도움을 거절하고 있다.
8	분노	H씨는 20대 여성이다. 지진으로 부모님이 실종된 상태이다. 자신을 취재하러 온 취재진에게 '찍지 말라'며 흥분하여 소리치고 있다.
9	서비스<br>불만	I씨는 30대 남성이다. 현재 아내가 실종되어 대피소 머무르고 있다. 대피소가 굉장히 낙후된 것에 대해서 행정 직원에게 불만을 제기하고 있다.
10	아동	J군은 K씨의 아들로 7세이다. 지진으로 매우 놀란 상태이며, 현재 상황에 대해서 귀찮을 정도로 엄마에게 질문하고 있다. 엄마가 잘 반응해 주지 않자 다른 곳에서 무슨 일이 벌어지는지 궁금해서 계속 돌아다니는 모습이며, 이런 행동으로 엄마에게 과도하게 야단을 맞고 있다.
11	보호자<br>스트레스	K씨는 30대 여성이다. 유치원생으로 보이는 자신의 아이에게 가만히 앉아 있으라고 소리를 지르고 짜증을 내는 모습을 보이고 있다.
12	분노	L씨는 30대 남성이다. L씨는 아직 자신의 가족이 도착하지 않았다며, 장비를 착용하고 직접 가족을 찾으러 가겠다고 하고 있다. 문 앞에서 관계자들이 저지하자 '당신들이 뭘 할 수 있냐'고 소리를 지르고 있다.
13	피해의식<br>편집증적	M씨는 50대 남성이다. 몸이 불편하다면서 기관에 의뢰를 해달라고 요청하였다. 심리요원이 의뢰를 위해 필요한 M씨의 인적사항을 물으니 M씨는 그걸 알아서 뭐하냐는 식으로 인상을 찡그리고, 개인정보 기입에 대해 과도하게 방어적인 태도를 보인다.
14	서비스<br>불만	N씨는 40대 여성이다. 옆자리에 있는 사람이 너무 시끄럽다며 자리를 바꿔달라고 요구하고 있다.
15	서비스<br>불만	O씨는 80대 남성이다. 본부에서 나누어준 안내문을 손에 들고 소리를 지르고 있다. 그는 안내문의 글씨가 너무 작다며 불만을 토로하고 있다.

16	멍한 상태	P씨는 50대 여성이다. 현재 실종자 가족 및 유가족들이 머물고 있는 쉼터에 들어와 있다. 제대로 먹지도 자지도 못한 채, 멍하게 구조 현장에서 딸이 구조되기만을 기다리고 있다.
17	분노	Q씨는 50대 남성이다. 지진으로 인한 건물 붕괴로 아내를 잃었다. 현재 실종자 가족 및 유가족들이 머무는 대피소에 머물고 있는데, '이 대피소도 붕괴될 수 있다'고 다른 사람들에게 소리치면서 더 안전한 건물로 가자고 선동하고 있다.
18	조력자	R씨는 40대 남성이다. 지진 현장에서 신속하게 구조되었으며 그다지 부상을 입지 않은 상태이다. 상대적으로 침착하게 재난 현장에 어떤 사람들이 있는지 파악하였으며, 다른 사람들의 상태에 대해서도 이야기를 해 줄 수 있는 준비가 되어 있다. 힘들어하는 주변 사람들에게 물을 가져다 주는 등 적극적으로 도움 행동을 하고 있다
19	노인	S씨는 70대 노인이다. 지진 현장에서 빠르게 구조되어 큰 상처 없이 근처 안전한 장소로 후송되었다. 기본 신상 정보를 알아내려고 질문을 드렸으나, 질문을 잘 알아듣지 못하는 모습을 보이고 있다.
20	서비스 불만	T씨는 30대 여성이다. 지진 현장에서 남편과 같이 구조되었다. 남편이 크게 상해를 입어 근처 병원으로 후송되었다고 한다. 그러나 지역 병원은 믿을 수 없다며 무조건 대학병원으로 후송해 달라고 소리치고 있다.

SIMULATION 2 화재 시나리오

오전 8시에 ○○시 △△동 한 아파트 8층에서부터 불길이 일기 시작하였습니다. 불길이 다른 층으로 번지면서 아파트 고층 전체가 불길에 휩싸였고, 화재가 발생한 아파트와 인접한 다른 아파트에도 불이 옮겨 붙어 피해규모가 커졌습니다. 화재 신고를 받고 온 소방대원들이 화재를 진압했고 다행히 불길이 쉽게 잡혔지만, 건축자재로부터 배출된 유독가스로 인해 200명이 넘는 사상자가 발생하였습니다. 한 순간에 집과 가족을 잃은 생존자들은 의료처치 후 △△초등학교 강당에 마련된 임시대피소에서 생활하고 있습니다.

아래의 역할을 천천히 살펴보시고, 각자가 맡을 역할을 선택해주십시오. 재난 피해 상황에 있는 대상자 역할을 설정하고, 시뮬레이션 준비시간 동안(약 10분) 선택한 역할을 어떻게 연기하면 좋을지 생각해 주시면 됩니다. 연습 동안 진지하게 참여해 주십시오. 잠시 후, 당신을 돕기 위해 몇몇의 사람들이 도착할 것입니다. 역할에 고착될 필요가 없고, 심리요원의 대처에 따라 자유롭게 반응하시면 됩니다.

생존자 사례

번호	주요문제	내용
1	분노	A씨는 30대 남성이다. A씨는 유독가스 때문에 자신의 아내가 죽었다며 시공업체가 어디냐고 소리 지르고 있고, 책임자 처벌을 요구하고 있다.
2	멍한 상태	B군은 중학생이다. 임시 대피소에서 생활하고 있는 내내 눈에 초점이 없고, 주변 사람들이 말을 걸어도 아무런 반응이 없다.
3	플래시백 수면장해	C씨는 30대 남성이다. 구출된 이후 어느 정도 안정이 되긴 하였으나, 불에 타고 있는 자신의 집과 심각한 부상을 입은 사람들이 구조되는 것이 계속해서 떠오른다고 보고하였다.
4	조력자	D씨는 50대 여성이다. D씨는 1주일 동안 임시 대피소 생활을 하면서 혼자 있는 다른 생존자들에게 말을 걸고 물과 담요를 챙겨주고 있다.
5	노인 두려움	E씨는 70대 여성이다. E씨는 화재가 나는 악몽에 시달려 잠에 들지 못하고 있으며, 대피소에도 불이 날지도 모른다며 소화기를 찾고, 자신의 자리는 대피소 입구 쪽에 만들어 달라고 요구하고 있다.
6	서비스 불만	F씨는 50대 남성이다. 몸이 불편하다면서 병원에서 무료로 치료받을 수 있는지 물었다. 심리요원이 무료 서비스를 받을 수 있는 곳을 안내하였지만, F씨는 그 병원이 싫다며 다른 병원을 요구하고 있다.

7	분노<br>편집증적<br>증상	G씨는 30대 남성이다. G씨는 대피소의 심리요원들이 자신을 비웃고, 자신에게만 부실한 음식을 줬다면서 도시락을 심리요원에게 던지고 있다.
8, 9	스트레스<br>청소년	H군과 I군은 중학생이다. 서로 장난을 치면서 웃다가 갑자기 몸싸움을 벌이면서, 말조심하라고 소리를 지르고 욕을 하고 있다.
10	두려움	J씨는 20대 여성이다. J씨는 눈물을 흘리면서 혼자 있는 것이 무섭다고 심리요원에게 도움을 요청하고 있다.
11	임산부	K씨는 30대 여성으로, 임신 3개월 정도 된 임산부이다. K씨는 대피소에서 복통을 호소하며 울고 있다.
12	보호자	K씨의 남편 L씨(30대)는 임산부를 위한 안내나 의료적 처치가 전혀 없고, 모든 사람을 동일한 장소에 한꺼번에 들어가게 하는 것이 부당하다며 심리요원에게 화를 내고 있다.
13	시각<br>장애인<br>두려움	M씨는 80대 남성이며 시각장애인이다. 현재 대피소에서 혼자 생활하고 있다. M씨는 이재민들이 무질서하게 물품을 들고 가는 소란스러운 소리에 놀란 표정을 지으며 바닥을 더듬으며 일어나려고 한다.
14	서비스<br>불만	N씨는 40대 남성이다. N씨는 지금까지 아무런 지원을 받지 않았다고 소리를 지르면서 생수, 음식, 담요 등의 물품을 마음대로 가져가려 하고 있다. 심리요원이 N씨에게 순서대로 물품을 나눠주겠다고 하였지만, N씨는 막무가내로 심리요원을 밀치며 물품을 챙기고 있다.
15	보호자가<br>없는<br>아동	O군은 8살 남자아이이다. 건물에서 구조되어 대피소로 오게 된 O군은 부모를 찾지 못해 울고 있다.
16	조력자	P씨는 30대 남성이다. 임시 대피소에서 생활하는 동안 심리요원을 도와 다른 생존자들을 위로해주고 있다.

17	분노	Q씨는 50대 여성이다. Q씨는 자신의 가족이 아직 도착하지 않았다면서, 직접 가족을 찾겠다고 대피소를 나가려고 하고 있다.
18	멍한 상태	R씨는 20대 남성이다. 임시 대피소에서 말없이 구석에 앉아 있다. 주변 사람들의 질문이나 요구에서 아무런 반응을 하지 않고 있다.
19	보호자 스트레스	R씨의 어머니인 S씨(40대)는 아들이 하루 종일 말이 없다며 불만을 이야기하고 있다. 이런 상태 때문에 계속 신경이 예민해 지고 가슴이 답답하다고 한다.
20	과각성	T씨는 60대 남성이다. 심리요원이 다가와서 인적사항을 물으니 ‘저리 가라’고 소리치고 있다.
