# Supplementary material for: A preliminary evaluation of the training effects of a didactic and simulation-based psychological first aid program in students and school counselors in South Korea
Source: PLoS One. 2017 Jul 17;12(7):e0181271. doi: 10.1371/journal.pone.0181271 (PMC5513559; doi:10.1371/journal.pone.0181271)
Supplement: S2 File — (PDF) [file pone.0181271.s005.pdf]

Supporting Information S2
Behavioral Checklists

1. Common Checklist for the Survivor

※ The following is a list of actions psychological personnel must do during psychological first aid.
- Fill in the ID of the psychological worker you met in the blank space on the right and evaluate their behavior towards you.

Common Checklist	psychological worker 1		psychological worker 2		psychological worker 3	
	YES	NO	YES	NO	YES	NO
When first meeting with the survivor, did they mention their name, affiliation, role, and purpose of the visit?						
Did they ask for permission to speak with you?						
Did they ask distinguishing information about the survivor (e.g., “What’s your name?” “How should I refer to you?” etc.)?						
Did they ask whether you needed anything?						
Did they ask how the situation has been until now?						
Did they provide help that would comfort a suffering person (e.g., providing information at the scene related to the rescue, or providing tissues, water, or drinks when needed)?						
Did they let the survivor know they were listening by nodding their head or positively responding to the survivor while they were speaking?						
Did they understand the survivor’s feelings and respond empathetically?						
If the survivor was searching for someone, did the psychological personnel member ask for distinguishing information about that person (e.g., “What is that person’s name?” “What are their physical features?” etc.)?						
Did they provide feedback about the things you did that were suitable in a disaster situation (e.g., quickly taking refuge, asking for help, helping others, etc.)?						
Did they answer the survivor’s questions based on all the facts?						

※ Please list any of the psychological worker’s actions not mentioned above that you thought were helpful, and actions you thought were not helpful.

Things that were helpful	Things that were not helpful

2. Common Checklist for Psychological Personnel

※ The following is a list of actions psychological personnel must do during psychological first aid.
- Please fill out the characteristics of the survivor you met in the blank space on the right, and evaluate their behavior towards you.

Common Checklist	Survivor 1		Survivor 2		Survivor 3	
	YES	NO	YES	NO	YES	NO
When meeting the survivor for the first time, did you introduce your name, affiliation, role, and purpose of visit?						
Did you ask permission to speak with them?						
Did you ask for distinguishing information about the survivor (e.g., "What is your name?" "How should I refer to you?" etc.)?						
Did you ask whether they needed anything?						
Did you ask how the situation has been until now?						
Did you provide help that would comfort a suffering person (e.g., providing information at the scene related to the rescue, or tissues, water, or drinks when needed?)?						
Did you let the survivor know you were listening by nodding your head or positively responding to them while they were speaking?						
Did you understand the survivor's feelings and respond empathetically?						
If the survivor was searching for someone, did you ask for distinguishing information about that person (e.g., "What is that person's name?" "What are their physical features?" etc.)?						
Did you provide feedback about the things the survivor did that were suitable in a disaster situation (e.g., taking refuge quickly, asking for help, helping others, etc.)?						
Did you answer the survivor's questions based on all the facts?						

※ Please list any of the psychological worker's actions not mentioned above that you thought were helpful, and actions you think were not helpful.

Things that were helpful	Things that were not helpful

3. Team-Building Checklist

※ The following is a list of actions psychological personnel must do in the team-building process of psychological first aid. From a leader's perspective, please evaluate whether our team carried out the actions below.

	Common Checklist	YES	NO
Team-building & mission	Did everyone share his/her disaster countermeasures experience?		
	Did everyone share their educational experience related to disasters?		
	Did the team determine the leader based on appropriate standards?		
	Was the basic spirit of psychological first aid or mission shared in the meeting process?		
Resources	Did the team distribute the space in a manner that was appropriate for the situation (situation headquarters, providing drinks, providing information, rest area for children, etc.)?		
	Did the team determine the materials they could use?		
	Did the team efficiently allocate materials in a manner that was appropriate to the situation of the rest area?		
Role	Did everyone share information about the role he/she took on?		
	Did those with similar roles gather and share the progress of their work?		
	Is there person who took on the central role that covers the entire process?		
Method	Was the process of dividing team roles voluntary/democratic?		
	Did the leader take initiative and perform a difficult role?		
Providing Information	Did the team share the basic practice of having to mention your name, affiliation, role, and purpose of the visit when meeting the survivor for the first time?		
	Did the team explain the situation concisely?		
	Did the team talk about the guidelines of psychological first aid thoroughly?		
Process Factors	At the beginning of providing service, did the leader or a responsible person give a briefing on the disaster situation?		

(Please observe during the simulation)	Were appropriate measures taken regarding the problem situation reported by team members (reporting activities, changing places, requesting information, etc.)?		
	Did the team determine the age and injury level of the survivor?		
	Did the team periodically check up on survivors who refused help?		
	Did the team control the media to protect the survivors?		

Other observations:

Strengths and weaknesses of the team-building process:

4. Individual Case Checklist Examples (Earthquake)

Survivor 1			
**A** is a 10-year-old girl. Her mother tries to comfort her, but A will not stop crying.			
Survivor 1 Checklist	**YES**	**NO**	Extra Points
Did the psychological worker ask for permission from the mother (Ms. B) to speak with **A**?			
Did the psychological worker attempt to converse with **A** at a level she could comprehend?			
Did the psychological worker direct **A** to a place where she could spend time safely (e.g., a place where she could play or draw)?			

Other observations:

____

Survivor 2			
Ms. B (in her 30s) is **A's** mother. She is doing her best to comfort her daughter, but her daughter will not stop crying. She is having a difficult time knowing what to do in this situation as it is also chaotic for her.			
Survivor 2 Checklist	**YES**	**NO**	Extra Points
Did the psychological worker ask for permission from the mother (Ms. B) to speak with **A**?			
Did the psychological worker explain to Ms. B the responses children can have to disaster situations based on their developmental stage?			
Did the psychological worker advise Ms. B on activities that could calm her daughter down?			

Other observations:

____

5. Individual Case Checklist Examples (Fire)

Survivor 1			
Mr. A is in his 30s. Mr. A says his wife died from toxic gas. He is yelling while asking where the construction company is and demands punishment for those responsible.			
Survivor 1 Checklist	YES	NO	Extra Points
Did the psychological worker take Mr. A to a quiet place where he could calm down?			
(If Mr. A continues to act hostile) Did the psychological worker seek help from other colleagues?			
Did the psychological worker listen so that Mr. A could sufficiently express his grievances and suffering?			
Did the psychological worker report Mr. A's demands to the control room?			
Did the psychological worker encourage Mr. A to find things he could do in the current situation?			
Did the psychological worker suggest that Mr. A engage in behaviors to take care of himself (e.g., eating, sleeping)?			
Did the psychological worker advice Mr. A on follow-up measures (e.g., when he could return for more information, or who could give him information)?			

Other observations:

____

Survivor 10			
Ms. J is in her 20s. While crying Ms. J says she is afraid to be alone and asks for help from the psychological personnel.			
Survivor 10 Checklist	**YES**	**NO**	Extra Points
Did the psychological worker check to see if there is a person who can help Ms. J?			
Did the psychological worker connect Ms. J with someone who could help her?			
(If Ms. J asks the psychological worker to stay with her). Did the psychological worker stay with her?			
Did the psychological worker talk with Ms. J about finding the small things she could do herself?			
(If it is difficult for the psychological worker to stay with her). Did the psychological worker inquire about someone who could stay with Ms. J?			

Other observations:

____

Behavioral Checklists (In Korean)

1. 생존자용 공통 체크리스트

※ 다음은 심리적 응급처치에서 심리요원이 기본적으로 해야 할 행동 목록입니다.

- 귀하가 만난 심리요원의 ID를 우측 칸에 기입하고, 그들이 귀하에게 한 행동을 평가해 주세요.

공통 체크리스트	심리요원 1		심리요원 2		심리요원 3	
	YES	NO	YES	NO	YES	NO
생존자와 첫 대면을 할 때, 이름, 소속, 역할, 방문 목적을 언급하였는가?						
이야기를 나눌 수 있는지 허락을 구하였는가?						
생존자를 식별할 수 있는 정보를 질문하였는가? (예: 성함이 어떻게 되시나요?, 어떻게 불러 드리면 좋을까요? 등)						
필요한 것이 있는지 질문하였는가?						
현재까지의 상황이 어땠는지 질문하였는가?						
고통스러워하는 사람에게 위로가 될 만한 것을 제공하였는가? (예, 현장에서 필요한 구조 관련 정보 제공, 필요시 휴지나 물, 음료 제공 등)						
생존자의 이야기에 고개를 끄덕이거나 호응을 하면서 경청하고 있음을 전달하였는가?						
생존자의 심정을 잘 헤아리며 공감적으로 반응하였는가?						
생존자가 찾는 사람이 있을 경우, 그 사람을 식별할 수 있는 정보를 질문하였는가? (예: 그 분의 성함은 어떻게 되시나요?, 그 분의 인상착의는 어떠한가요? 등)						
생존자에게 현장상황에서 적절하게 행동한 부분에 대해서 피드백을 제공하였는가? (예: 신속하게 대피하신 것은~, 도움을 요청한 것은~, 다른 사람에게 도움을 준 것은~ 등)						
생존자의 질문에 대해 사실관계에 기반 하여 대답하였는가?						

※ 위 목록에 없지만, 심리요원의 행동 중 도움이 되었다고 생각되는 부분과 도움 되지 않았다고 생각되는 부분이 있다면 작성해 주세요.

도움 된 부분	도움 되지 않은 부분

2. 심리요원용 공통 체크리스트

※ 다음은 심리적 응급처치에서 심리요원이 기본적으로 해야 할 행동 목록입니다.

- 귀하가 만났던 생존자의 특징을 우측 칸에 기입하고, 그들에게 귀하가 했던 행동을 평가해 주세요.

공통 체크리스트	생존자 1		생존자 2		생존자 3	
	YES	NO	YES	NO	YES	NO
생존자와 첫 대면을 할 때, 이름, 소속, 역할, 방문 목적을 언급하였는가?						
이야기를 나눌 수 있는지 허락을 구하였는가?						
생존자를 식별할 수 있는 정보를 질문하였는가? (예: 성함이 어떻게 되시나요?, 어떻게 불러 드리면 좋을까요? 등)						
필요한 것이 있는지 질문하였는가?						
현재까지의 상황이 어땠는지 질문하였는가?						
고통스러워하는 사람에게 위로가 될 만한 것을 제공하였는가? (예, 현장에서 필요한 구조 관련 정보 제공, 필요시 휴지나 물, 음료 제공 등)						
생존자의 이야기에 고개를 끄덕이거나 호응을 하면서 경청하고 있음을 전달하였는가?						
생존자의 심정을 잘 헤아리며 공감적으로 반응하였는가?						
생존자가 찾는 사람이 있을 경우, 그 사람을 식별할 수 있는 정보를 질문하였는가? (예: 그 분의 성함은 어떻게 되시나요?, 그 분의 인상착의는 어떠한가요? 등)						
생존자에게 현장상황에서 적절하게 행동한 부분에 대해서 피드백을 제공하였는가? (예: 신속하게 대피하신 것은~, 도움을 요청한 것은~, 다른 사람에게 도움을 준 것은~ 등)						
생존자의 질문에 대해 사실관계에 기반 하여 대답하였는가?						

※ 위 목록에 없지만, 심리요원의 행동 중 도움이 되었다고 생각되는 부분과 도움 되지 않았다고 생각되는 부분이 있다면 작성해 주세요.

도움 된 부분	도움 되지 않은 부분

3. 팀빌딩 체크리스트

※ 다음은 심리적 응급처치의 팀빌딩 과정에서 심리요원들이 해야 할 행동 목록입니다. 우리 팀이 아래의 사항을 수행했는지 리더의 입장에서 평가해 주십시오.

	공통 체크리스트	YES	NO
팀빌딩 & 미션	각자의 재난 대응 경험을 공유하였는가?		
	각자의 재난 관련 교육 경험을 공유하였는가?		
	적절한 기준에 따라 리더를 결정하였는가?		
	심리적 응급처치의 기본 정신이나 미션이 무엇인지 회의 과정에서 공유하였는가?		
자원	주어진 공간을 상황(상황본부, 음료제공, 정보제공, 아이들 쉼터 등)에 맞게 구분하였는가?		
	활용할 수 있는 물품을 파악하였는가?		
	물품을 쉼터 상황에 맞게 효율적으로 배분하였는가?		
역할	각자 맡을 역할에 대해 정보를 공유하였는가?		
	역할이 유사한 사람들끼리 모여서 업무 진행 내용을 공유하였는가?		
	전체 과정을 총괄하는 허브 역할을 할 사람이 존재하는가?		
방식	팀 역할을 나누는 과정이 자발적/민주적이었는가?		
	리더가 솔선하여 어려운 역할을 맡았는가?		
정보제공	생존자와 첫 대면을 할 때, 이름, 소속, 역할, 방문 목적을 언급해야 한다는 기본 수칙을 공유하였는가?		
	상황에 대해서 간략하게 설명하였는가?		
	심리적 응급처치의 지침에 대해서 충분히 이야기를 나누었는가?		
과정변인 (시뮬레이션을	서비스를 제공하는 초반부에 리더 또는 책임감 있는 사람이 재난 상황에 대해 브리핑 하였는가?		

하는 동안 관찰하세요)	팀원이 보고한 문제 상황(취재활동, 자리 이동, 정보 요구 등)에 대해 적절한 조치를 취하였는가?		
	전체 생존자의 연령, 피해 정도를 전체적으로 파악하였는가?		
	도움을 거절한 생존자에게 일정 간격으로 다시 상태를 확인하는 모습을 보였는가?		
	생존자를 보호하기 위해 언론을 통제하였는가?		

기타 관찰 사항:

팀 빌딩 과정의 장점 / 단점:

4. 개별사례 체크리스트 예시(지진)

생존자 1			
A양은 10살 여자아이이다. A양의 어머니가 계속해서 아이를 달래고 있지만 A양은 울음을 그치지 않는다.			
생존자 1 체크리스트	**YES**	**NO**	추가점수
심리요원은 어머니(B씨)에게 A양과 심리요원이 대화를 해도 되는지 허락을 구하였는가?			
심리요원은 A양과 눈높이를 맞추며 대화를 시도하였는가?			
심리요원은 A양이 안전하게 시간을 보낼 수 있는 장소를 안내하였는가?<br>(예: 놀이나 그림 그리기가 가능한 곳)			

기타관찰사항: ____________________

생존자 2			
B씨는 A양의 어머니이다(30대). 딸을 달래려 최선을 다하고 있지만 아이가 울음을 그치지 않고 있다. B씨는 본인도 혼란스러운 상황에서 아이에게 어떻게 대해야 할지 몰라 힘들어 하는 상황이다.			
생존자 2 체크리스트	**YES**	**NO**	추가점수
심리요원은 B씨(어머니)에게 A양과 심리요원이 대화를 해도 되는지 허락을 구하였는가?			
심리요원은 B씨에게 발달 단계에 따라 재난 후에 아동이 보일 수 있는 반응에 대해서 설명하였는가?			
심리요원은 B씨(어머니)에게 A양을 진정시킬 수 있는 활동들에 대해서 안내했는가?			

기타관찰사항: ____________________

5. 개별사례 체크리스트 예시(화재)

생존자 1			
A씨는 30대 남성이다. A씨는 유독가스 때문에 자신의 아내가 죽었다며 시공업체가 어디냐고 소리 지르고 있고, 책임자 처벌을 요구하고 있다.			
생존자 1 체크리스트	**YES**	**NO**	추가점수
심리요원은 A씨가 진정할 수 있도록 조용한 장소로 A씨를 데려갔는가?			
(A씨가 계속 흥분된 모습을 보인 경우) 심리요원은 다른 동료에게 도움을 구하였는가?			
심리요원은 A씨가 억울함과 고통을 충분히 표현할 수 있도록 들어주었는가?			
심리요원은 A씨의 요구사항을 상황실에 보고하였는가?			
심리요원은 A씨가 현재 이 상황에서 할 수 있는 일을 찾도록 격려하였는가?			
심리요원은 A씨가 진정할 수 있도록 조용한 장소로 A씨를 데려갔는가? (예: 식사, 수면)			
(A씨가 계속 흥분된 모습을 보인 경우) 심리요원은 다른 동료에게 도움을 구하였는가? (예: 언제까지 돌아와서 알려드릴 수 있는지, 누가 알려드릴 수 있는지 등)			

기타관찰사항: ____________________

생존자 10			
J씨는 20대 여성이다. J씨는 눈물을 흘리면서 혼자 있는 것이 무섭다고 심리요원에게 도움을 요청하고 있다.			
생존자 10 체크리스트	**YES**	**NO**	추가점수
심리요원은 J씨에게 도움을 줄 수 있는 사람이 있는지 확인하였는가?			
심리요원은 J씨에게 도움을 줄 수 있는 사람을 연결시켜주었는가?			
(J씨가 심리요원에게 같이 있어달라고 요구한다면) 같이 있어주었는가?			
심리요원은 J씨가 작은 일이라도 스스로 할 수 있는 일을 찾을 수 있도록 이야기를 나누었는가?			
(심리요원이 함께 있는 것이 힘든 경우) 심리요원은 J씨와 함께 있어 줄 사람을 알아보았는가?			

기타관찰사항: ____________________
