# Supplementary material for: A preliminary evaluation of the training effects of a didactic and simulation-based psychological first aid program in students and school counselors in South Korea
Source: PLoS One. 2017 Jul 17;12(7):e0181271. doi: 10.1371/journal.pone.0181271 (PMC5513559; doi:10.1371/journal.pone.0181271)
Supplement: S3 File — (PDF) [file pone.0181271.s006.pdf]

Supporting Information S3
PFA Knowledge Tests

PRE-TEST

■ The following covers psychological first aid (PFA).

1. Which of the following should <u>not</u> take place in the rescue phase?

① Restorative environment monitoring

② Determining the survivor's current state and needs

③ Connected activity with a service group

④ Triage (selective evaluation)

2. Which of the following is the most correct explanation of PFA?

① Professional psychological therapy provided at the beginning of a disaster to prevent after effects.

② Carrying out PFA to prevent serious mental problems that all survivors may encounter.

③ Carrying out PFA onsite, or in a location close to the site, away from traditional treatment facilities.

④ Psychological personnel must provide help above their own expertise and limitations.

3. Which of the following is the most accurate example of carrying out PFA?

① You observed the situation after arriving at the scene.

② Even though you were unsure of the accuracy, you relayed information to the survivor to set them at ease.

③ You provided counsel regarding the survivor's grief reaction.

④ You relayed the content of the discussion with the survivor to the person in charge.

4. Which of the following is the most accurate example of carrying out PFA?

① You offered words of comfort such as "everything will be okay."

② You maintained eye contact with the translator while conversing with a foreign survivor.

③ You clearly enunciated in a low tone while speaking to a survivor with poor hearing.

④ You helped a visually impaired person by first taking hold of their arm.

5. Which of the following is most inappropriate response when attempting to provide information to a survivor as part of PFA?

① Providing information about post-traumatic stress disorder.

② Providing information about the current disaster state and situation in the future.

③ Providing information about general stress responses.

④ Providing information about coping skills.

6. Regarding the psychological personnel's response after they finish psychological support at the earthquake zone and return to work, which of the following is the most appropriate in coping as a colleague?

Psychological personnel: I saw a deceased person at the rescue scene. I imagine the fear that person must have felt inside the building, but it's too difficult. How could this have happened? It was truly horrible.

① A: True, but what can we do? That person's family and friends must be having a more difficult time than you. Just try and hang in there.
② B: It was awful for me too. But things will work out.
③ C: Yes. All of the people who assisted at the scene have a drink together and forget about it.
④ D: It was really difficult, right? When you had this kind of stress in the past, how did you overcome it?

7. Which of the following is the <u>most inappropriate</u> response for psychological personnel regarding the family of the deceased?
① Before children enter school, explain the meaning of death to them realistically.
② Use the specific name of the deceased family member.
③ Consider the safety of the survivor who wants to be alone and prepare a location as private as possible.
④ Immediately comfort the survivors who have been informed of the death.

8. Which of the following is the most <u>inappropriately</u> applied stabilization technique for emotionally overwhelmed survivors?
① Having them close their eyes.
② Having them exhale deeply.
③ Having them feel their feet touch the ground.
④ Having them say the colors of objects in their vicinity.

9. Which of the following is the most suitable behavior for psychological personnel?
① You devotedly continue to try and help because you feel guilty about taking breaks.
② You limit working hours to not exceed 12 hours a day.
③ You go out for drinks with your colleagues often to forget about difficult scenes you encountered.
④ You do your best with a sense of duty that tells you only you can do it.

10. List 5 self-care activities to prevent vicarious trauma or exhaustion.
① ______________________________
② ______________________________
③ ______________________________
④ ______________________________
⑤ ______________________________

POST TEST

■ The following content is about psychological first aid (PFA).

1. Which of the following is the most correct PFA for Mr. A, one of the psychological personnel, to perform following a disaster?

① He went with a friend to the disaster scene to help in any way possible.
② He recognized the things he did not know about the survivor's questions regarding compensation.
③ He asked survivors, "What symptoms are you currently experiencing?"
④ He leaves his family life and occupation behind, and stays at the disaster scene for 1 month.

2. Which of the following is the most inappropriate intervention for emotionally overwhelmed children?

① Explain that these situations can be surprising for adults also.
② Give advice to the parents for calming the child while pointing out their mistakes.
③ Place the child next to the parents and first have them calm the child.
④ Perform relaxation techniques with the child if the parents are unable to bear the situation.

3. Which of the following is the most inappropriate behavior for the first meeting with a survivor?

① Continue speaking to a survivor who refuses help.
② Report to an institution that a child survivor has been abused.
③ Respect the choice of the survivor who rejects help.
④ Reveal your name and affiliation to the survivor and ask if it is okay to have a conversation.

4. Which of the following is the most appropriate response from psychological personnel in performing PFA with a child who lost their parents in a plane accident?

① "I think I know how you feel having lost your parents."
② "Do you have any relatives, brothers, or sisters from whom you can get help?"
③ "This is a very sad situation, but you're lucky to have an older sister."
④ "Your parents would be so proud if they saw how you're holding up."

5. Which of the following is the most appropriate response from psychological personnel in PFA?

① Have the survivor get the latest information related to the disaster from the Internet or TV.
② Comfort the survivor and relieve their sense of guilt by saying, "Maybe this was for the best."
③ Explain the danger to the survivor who says they will go out and find their missing family and dissuade them from doing so.
④ Explain, in detail, the situation to the child wondering about the current disaster state.

6. Which of the following is the most appropriate coping method in a situation where a body needs to be identified?

① Explain, in detail, to the family of the deceased the state in which the body was found and the suffering they went through at the time of death.
② Do not inform the child if they ask specifically about the corpse of their deceased parent(s).
③ Tell the family of the deceased about coping methods when they are informed of the death.
④ Try your best to answer religious or spiritual questions the survivor might have.

7. Which of the following is the most appropriate intervention for survivors in PFA?

① Ask specific questions about the disaster experience and collect sufficient information for follow-up measures.
② Consider all of the solutions for the several concerns the survivor mentions.
③ Arrange a small group in which survivors can form relationships with each other.
④ As a member of the psychological personnel, take action on behalf of the survivor in problem solving.

8. Which of the following is the furthest away from a situation requiring psychiatric referral?

① When there is an alcohol or drug use problem
② When existing mental problems are worsening
③ When the survivor says they are having nightmares 2 weeks after the accident
④ When the survivor requests help

9. Which of the following is not a measure an institution needs to take in order to protect psychological personnel?

① Job rotation based on the level of work stress
② Recruiting a few seasoned experts that can provide PFA
③ Giving leave to psychological personnel who have participated over a certain amount of hours
④ Implementing periodic evaluations of personnel with high levels of stress

10. List 5 self-care activities to prevent vicarious trauma or exhaustion.

⑥ ______________________________
⑦ ______________________________
⑧ ______________________________
⑨ ______________________________
⑩ ______________________________

PFA Knowledge Tests (In Korean)

PRE-TEST

■ 다음은 심리적 응급처치(PFA)에 관한 내용입니다.

1. 다음 중 구출단계에서 해야 하는 개입과 거리가 가장 먼 것은?

① 회복환경 모니터링
② 생존자의 현재 상태와 욕구 파악
③ 봉사단체와 연계된 활동
④ 트리아지(선별 평가)

2. 다음 중 심리적 응급처치에 대한 가장 옳은 설명은?

① 재난 초기에 후유증을 예방하기 위한 전문적인 심리치료이다.
② 모든 생존자가 심한 정신적 문제를 겪을 수 있으므로, 이를 예방하기 위해 실시된다.
③ 전통적인 치료기관에서 벗어나 현장 또는 현장에서 가까운 곳에서 실시된다.
④ 심리요원은 자신의 전문성과 한계 이상으로 생존자에게 도움을 줘야 한다.

3. 다음 중 심리적 응급처치를 가장 올바르게 실시한 것은?

① 현장에 도착한 후 우선 상황을 관찰했다.
② 확실하지 않지만 생존자를 안심시키기 위해 정보를 전달했다.
③ 생존자의 애도반응에 대한 상담을 제공했다.
④ 생존자와 나눈 이야기를 책임자에게 전달했다.

4. 다음 중 심리적 응급처치를 가장 올바르게 실시한 것은?

① "다 잘 될 거예요"라고 위로의 말을 건넸다.
② 외국인 생존자와 대화하면서 통역사를 쳐다보면서 대화했다.
③ 청력이 좋지 않은 생존자에게 낮은 톤으로 명확하게 발음했다.
④ 시각장애인의 팔을 잡고 먼저 부축해주었다.

5. 다음 중 심리적 응급처치의 일부로 생존자들에게 정보를 제공하려고 할 때, 가장 부적절한 반응은?

① 외상 후 스트레스 장애에 대한 정보
② 현재까지 재난 상황과 앞으로의 상황에 대한 정보
③ 일반적인 스트레스 반응에 대한 정보
④ 대처 기술에 대한 정보

6. 지진 현장에서 심리지원을 마치고 직장에 복귀한 심리요원의 반응이다. 다음 중 동료로서 가장 적절한 대처는?

심리요원: 구조 현장에서 사망자를 봤어요. 그 사람이 그 건물 안에서 겪었을 공포를 상상하니 너무 힘들어요. 어떻게 이런 일이 일어날 수 있죠? 너무 끔찍했어요.

① A: 맞아요. 그래도 어쩌겠어요. 그 사람의 가족과 지인은 당신보다 더 힘들 거예요. 조금만 더 힘내세요.
② B: 저도 너무 끔찍했어요. 그래도 잘 해결될 거예요.
③ C: 네. 현장 지원을 다녀온 사람들끼리 술 한 잔하고 잊어버립시다.
④ D: 많이 힘들었죠? 이런 스트레스가 있었을 때 전에는 어떤 방식으로 극복하셨나요?

7. 다음 중 가족이 사망한 생존자에 대한 심리요원의 반응 중 가장 부적절한 것은?
① 취학 전 아동에게 죽음의 의미에 대해 사실적으로 알려준다.
② 사망한 가족의 이름을 구체적으로 사용한다.
③ 혼자 있고 싶어 하는 생존자에게 안전을 고려하여 가능한 사적인 장소를 마련해준다.
④ 사망 소식을 들은 생존자를 즉시 위로한다.

8. 다음 중 정서적으로 압도된 생존자에게 안정화 기법을 가장 부적절하게 적용한 것은?
① 눈을 감도록 한다.
② 깊게 숨을 내쉬도록 한다.
③ 발이 땅에 닿는 느낌을 느껴보도록 한다.
④ 주변에 보이는 물건의 색깔을 말하게 한다.

9. 다음 중 심리요원의 행동으로 가장 바람직한 것은?
① 휴식을 취하는 것이 미안해서 헌신적으로 돕는다.
② 근무 시간이 하루 12시간을 넘지 않도록 제한한다.
③ 힘들었던 장면을 잊기 위해 동료들과 자주 술자리를 가진다.
④ 나만 할 수 있는 일이라는 사명감을 갖고 최선을 다한다.

10. 대리외상이나 소진을 예방하기 위한 자기 돌봄 활동 5가지를 적으시오.
① ______________________
② ______________________
③ ______________________
④ ______________________
⑤ ______________________

POST TEST

■ 다음은 심리적 응급처치(PFA)에 관한 내용입니다.

1. 다음 중 재난 발생 후, 심리요원 A씨가 행한 심리적 응급처치로 가장 옳은 것은?

① 뭐든 도움이 되고자, 재난 현장에 친구와 둘이서 갔다.

② 보상에 관한 생존자의 질문에 모르는 부분임을 인정했다.

③ 생존자에게 "현재 어떤 증상을 경험하고 있나요?"라고 질문했다.

④ 가정생활과 생업을 뒤로 한 채, 한 달째 현장을 지키고 있다.

2. 다음 중 정서적으로 압도된 아동에게 가장 부적절한 개입은?

① 아동에게 이런 상황에서 어른들도 놀랄 수 있음을 설명한다.

② 아동을 진정시키고 있는 부모에게 잘못을 지적하면서 조언한다.

③ 아동을 부모 곁으로 데려가 부모가 우선 아동을 진정시키도록 한다.

④ 부모가 상황을 감당하지 못할 경우, 아동에게 이완 기법을 시행한다.

3. 다음 중 생존자와의 첫 대면에서 가장 부적절한 행동은?

① 도움을 거부하는 생존자에게 계속 말을 건다.

② 아동 생존자가 학대 받은 사실을 기관에 보고한다.

③ 도움을 거절하는 생존자의 의사를 존중한다.

④ 생존자에게 이름과 소속을 밝히고 이야기를 나눌 수 있는지 묻는다.

4. 다음 중 비행기 사고로 부모를 잃은 청소년과의 심리적 응급처치에서 심리요원의 반응 중 가장 적절한 것은?

① 부모를 잃은 네 기분이 어떤 지 알 것 같아.

② 혹시 도움을 받을 수 있는 친척이나 형제, 자매가 있니?

③ 지금 이 상황이 너무 슬프지만, 그나마 누나가 있어서 불행 중 다행이야.

④ 고인들께서 이렇게 잘 견디고 있는 모습을 본다면, 대견해하실 것 같아.

5. 다음 중 심리적 응급처치에서 심리요원의 가장 적절한 반응은?

① 인터넷이나 TV를 통해 생존자가 재난 관련 최신 정보를 얻도록 한다.

② "아마도 이게 최선이었을 거예요"라고 위로하며 생존자의 죄책감을 덜어준다.

③ 실종 가족을 찾겠다고 나서는 생존자에게 위험성을 알리고 만류한다.

④ 현재 재난 상황에 대해 궁금해 하는 어린 아동에게 상황을 자세하게 설명한다.

6. 다음 중 시신 확인이 필요한 상황에서 가장 적절한 대처는?

① 유가족에게 사망자의 발견 상태와 사망 당시 고통에 대해 상세하게 알려준다.

② 아이들이 사망한 부모의 사체에 대해 구체적으로 물을 경우 알려주지 않는다.

③ 사망 소식을 전해들은 유가족 옆에서 도움이 되는 대처 방법에 대해 말해준다.

④ 생존자가 종교적, 영적 질문을 할 경우, 나름대로 답을 하려고 노력한다.

7. 다음 중 심리적 응급처치에서 생존자에 대한 가장 적절한 개입은?

① 재난 경험에 대해 자세하게 질문하여 후속 조치를 위한 충분한 정보를 수집한다.
② 생존자가 언급한 여러 가지 걱정에 대한 해결책을 한꺼번에 고려한다.
③ 생존자들이 서로 관계를 형성하도록 소규모 집단을 주선한다.
④ 심리요원이 생존자 대신 문제 해결을 위한 행동을 취한다.

8. 다음 중 정신과 의뢰가 필수적인 상황과 가장 거리가 먼 것은?

① 술이나 약물 사용 문제가 있을 때
② 기존의 정신적 문제가 악화될 때
③ 사고 2주 후 악몽을 꾼다고 호소할 때
④ 생존자 본인이 의뢰를 요구할 때

9. 다음 중 심리요원을 보호하기 위해 기관이 취해야 할 조치가 아닌 것은?

① 업무의 스트레스 수준에 따라 순환 근무
② 심리적 응급처치를 제공할 수 있는 소수 정예의 전문 인력 모집
③ 일정 기간 이상 참여한 심리요원에게 휴가 부여
④ 스트레스가 높은 요원에 대한 주기적인 평가 실시

10. 대리외상이나 소진을 예방하기 위한 자기 돌봄 활동 5가지를 적으시오.

① ______________________________
② ______________________________
③ ______________________________
④ ______________________________
⑤ ______________________________
