# Supplementary material for: A preliminary evaluation of the training effects of a didactic and simulation-based psychological first aid program in students and school counselors in South Korea
Source: PLoS One. 2017 Jul 17;12(7):e0181271. doi: 10.1371/journal.pone.0181271 (PMC5513559; doi:10.1371/journal.pone.0181271)
Supplement: S4 File — (PDF) [file pone.0181271.s007.pdf]

Supporting Information S4
Perceived Willingness, Preparedness, and Confidence

※ The following are questions regarding how you would act and feel in the event of a future disaster. Please check the box you feel most suits you.

1. If any of the following disasters were to occur in the community you live, would you be **willing** to provide psychological assistance (e.g. psychological first aid) to survivors?

No.	Disaster Type	Degree of Willingness				
		0<br>Not at all	1<br>Not really	2<br>Somewhat	3<br>Yes	4<br>Very much so
1	**Earthquake**					
2	**Fire**					
3	**Typhoon**					
4	**Explosion**					

2. If any of the following disasters were to occur in the community you live, would you be **prepared** to provide psychological assistance (e.g. psychological first aid) to survivors?

No.	Disaster Type	Level of Preparation				
		0<br>Not at all	1<br>Not really	2<br>Somewhat	3<br>Yes	4<br>Very much so
1	**Earthquake**					
2	**Fire**					
3	**Typhoon**					
4	**Explosion**					

3. If any of the following disasters were to occur in the community you live, would you be **confident (competent)** in providing psychological assistance (e.g. psychological first aid) to survivors?

No.	Disaster Type	Level of Confidence (Competence)				
		0<br>Not at all	1<br>Not really	2<br>Somewhat	3<br>Yes	4<br>Very much so
1	**Earthquake**					
2	**Fire**					
3	**Typhoon**					
4	**Explosion**					

Perceived Willingness, Preparedness, and Confidence
(In Korean)

※ 다음은 미래의 재난발생 시 귀하가 어떻게 행동하고 느끼실 지에 관한 질문들입니다. 가장 적합하다고 생각되는 칸에 체크해 주십시오.

1. 만일 귀하가 거주하는 지역사회에서 다음과 같은 재난이 발생한다면 현장에서 생존자들에게 심리적 지원(예: 심리적 응급처치)을 제공할 **의향**이 있으십니까?

번호	재난 내용	의향의 정도				
		0<br>전혀 없음	1<br>별로 없음	2<br>보통	3<br>있음	4<br>상당히 있음
1	**지진**					
2	**화재**					
3	**태풍**					
4	**폭발사고**					

2. 만일 귀하가 거주하는 지역사회에서 다음과 같은 재난이 발생한다면 현장에서 생존자들에게 심리적 지원(예: 심리적 응급처치)을 제공할 **준비**가 되어 있습니까?

번호	재난 내용	준비 정도				
		0<br>전혀 안되어 있음	1<br>아직 부족함	2<br>보통 정도임	3<br>되어 있음	4<br>상당히 잘 되어 있음
1	**지진**					
2	**화재**					
3	**태풍**					
4	**폭발사고**					

3. 만일 귀하가 거주하는 지역사회에서 다음과 같은 재난이 발생한다면 현장에서 생존자들에게 심리적 지원(예: 심리적 응급처치)을 제공할 **자신감(유능감)**이 있습니까?

번호	재난 내용	자신감(유능감) 정도				
		0<br>전혀 없음	1<br>아직 부족함	2<br>보통 정도임	3<br>있음	4<br>상당히 있음
1	**지진**					
2	**화재**					
3	**태풍**					
4	**폭발사고**					
